# Effect of perinatal depression on birth and infant health outcomes: a systematic review and meta-analysis of observational studies from Africa

**DOI:** 10.1186/s13690-022-00792-8

**Published:** 2022-01-20

**Authors:** Abel Fekadu Dadi, Temesgen Yihunie Akalu, Haileab Fekadu Wolde, Adhanom Gebreegziabher Baraki

**Affiliations:** 1 Department of Epidemiology and Biostatistics, Institute of Public Health, College of Medicine & Health Sciences, University of Gondar, Gondar, Ethiopia; 2 School of Public Health, College of Medicine and Public Health, Flinders University, Bedford Park, Australia

**Keywords:** Antenatal depression, Perinatal depression, Adverse birth outcomes, Adverse infant health outcomes, Systematic review, Meta-analysis

## Abstract

**Background:**

Antenatal depression is associated with intrauterine growth retardation, preterm birth, and low birth weight. Infants born to mothers with postnatal depression also may suffer from malnutrition and other health problems. Even though there are few single studies conducted so far, a systematic review of these studies is highly important to highlight the effect of antenatal and perinatal depression on adverse birth and infant health outcomes in Africa.

**Methods:**

We used the Preferred Report Items for Systematic Review and Meta-analysis (PRISMA) when conducting this study. Databases like CINAHL (EBSCO), MEDLINE (via Ovid and PubMed), PsycINFO, Emcare, Psychiatry Online, and Scopus were searched. In addition, Google Scholar and references from a list of eligible studies were explored. We included good quality observational studies based on Newcastle Ottawa Scale which are published in the English language between 2007 and 2018.  Heterogeneity and publication bias were assessed. Meta-analysis with a random effect model was employed to determine the pooled effect sizes with a 95% confidence interval. The review protocol is registered in PROSPERO (CRD42018106714).

**Result:**

We found three studies (1511 participants) and 11 studies (22,254 participants) conducted on the effect of antenatal depression on birth outcomes and perinatal depression on adverse infant health outcomes, respectively. The overall risk of having adverse birth outcomes was 2.26 (95% CI: 1.43, 3.58) times higher among pregnant mothers with depression. The risk of preterm birth and low birth weight was 1.77 (95% CI: 1.03, 3.04) and 2.98 (95% CI: 1.60, 5.55) respectively. Similarly, the risk of having adverse infant health outcomes namely malnutrition and febrile illness was 1.61 (95% CI: 1.34, 1.95) times higher among mothers who had perinatal depression.

**Conclusions:**

We have found a significant association between antenatal depression and adverse birth outcomes, low birth weight and preterm birth. Similarly, a significant effect of perinatal depression on adverse infant health outcomes namely, malnutrition, and febrile illnesses was observed. The findings highlight that it is time to integrate mental health services with routine maternal health care services to improve birth outcomes and reduce infant morbidity.

**Supplementary Information:**

The online version contains supplementary material available at 10.1186/s13690-022-00792-8.

## Background

Depression is the third leading cause of disease burden worldwide and is predicted to become the second leading cause of the global disease burden by the year 2020 [1]. Women are twice as likely to be diagnosed with depression than men [2]. Women of child-bearing age frequently suffer from major depression [3]. Depression is also one of the most prominent mental health disorders in women following delivery [4]. Depression during pregnancy and the postnatal period, which is referred as perinatal depression onwards in this paper is a non-psychotic depressive episode that challenges both mothers and the health care providers [5, 6]. The prevalence of depression ranges from 11.3 to 19.6% during pregnancy and 9.6% to 24.3% during the postpartum period [7–10].

Depression during pregnancy and post-partum negatively affect both maternal and fetal health [4, 11]. According to studies from developed countries, the effect could be direct to the fetus or indirectly through unhealthy maternal behaviors arising from depression [3]. Some of the effects on the newborn are low birth weight [12–16], intra-uterine growth restriction (IUGR), preterm delivery [17], low Apgar score [18], and impaired infant growth that includes shorter length and lighter weight [5, 19]. Similarly, based on primary studies from developed countries, postnatal depression could cause infant ill-treatment [20], alcohol abuse leading to poor infant care [21], infant irritability [22, 23], impaired cognitive function [24], malnutrition [25], and infant death [26]. However specific studies on this area are limited or lacking in low-income countries.

The mechanism by which depression causes damage to the fetus could be due to the release of stress hormones such as cortisol and catecholamine [27], which in turn affect fetal growth [19] and gestational age [28]. The other explanation could be maternal malnutrition aroused by lack of appetite [29]. Poor dietary patterns by the mother due to depression could be also another mechanism by which the effect of depression on fetal growth could be explained [18].

Even though perinatal depression is a major public health problem, most of the literature and reviews were focused on the post-delivery period and were conducted to estimate its prevalence and risk factors. Therefore, limited information is available on the effect of perinatal depression on birth and infant health outcomes in Africa. The findings from individual studies also had inconsistent findings that could result in information dilemmas and affect public health interventions. In addition, perinatal depression has become more prevalent in African countries than in any other middle- and low-income countries, and we hypothesized that its effect would be also different. The findings from such specific settings will make policymakers and programmers more reactive as it clearly shows the problem in the context of Africa. Moreover, Africa is least developed continent with underdeveloped health infrastructure; therefore, pooling the effect of perinatal depression in Africa with that of developed countries would underestimate the problem.

## Method

### Searching strategy and inclusion criteria

We followed the Preferred Report Items for Systematic Review and Meta-analysis (PRISMA) when conducting this study [30]. We have done a thorough systematic search of the literature in CINAHL (EBSCO), MEDLINE (via Ovid and PubMed), PsycINFO, Emcare, Psychiatry Online, Scopus databases, and using search engines such as Google, Google Scholar, and following snowball search strategy (Additional file 1).

Our first review question was “Is there an association between antenatal depression and adverse birth outcomes such as low birth weight and preterm birth? The second review question was “Does perinatal depression (depression during pregnancy and postpartum) have an association with adverse infant health outcomes such as malnutrition and illnesses?”

Observational studies conducted in Africa from 2007 to 2018, which were written in English-language, used standardized tools for screening depression, and measured infant health outcomes following the standardized guideline for Integrated Management of New-born and Childhood Illness (IMNCI) were included. We included studies published after 2007 for information is dynamic and considering the most updated pieces of evidence would make the review result more homogeneous, up-to-date, and help us to have a recent image of the problem in Africa.

### Outcome definitions

Primary studies that used the following definition of outcome were considered. Low birth weight, birth weight less than 2500 g; Preterm birth, birth occurring after 20 complete weeks and before 37 complete weeks of gestation. Malnutrition was also considered when the Middle-Upper Arm-Circumference (MUAC) of the infant is less than 110 mm for infants 6–12 months and a weight-for-length ≥ − 2 Z-scores of the WHO Child Growth Standards median for infants under six months old.

Studies conducted among specific risk populations, such as HIV/AIDS patients, patients with chronic illnesses, and migrant populations were excluded. Following systematic searching, eligible studies were exported to Endnote version 7, and duplicates were removed. Studies that fulfilled the inclusion criteria through their title and abstract review were considered for full-text review. The full text of the included studies was reviewed by two reviewers (AFD, AGB). Disagreements were resolved by discussion and/or with the assistance of the third reviewer (HFW).

### Quality assessment and data extraction

The quality of evidence and the risk of bias were assessed by AFD and AGB using the Newcastle-Ottawa Scale (NOS) [31]. Based on the NOS, we had three classifications of studies: Good quality, studies with ≥7 points; Fair quality, studies with 2–6 points; Poor quality, studies with less than or equal to one [32]. All studies fulfilled the criterion for good quality and were included in the final review and meta-analysis. Structured data abstraction form was prepared and the following information was collected from each primary study: Name of the author, year of publication, country, country income, study setting, study design, sample size, time by which depression screening conducted, the tool used to screen depression, and effect sizes OR/RR/HR with 95% confidence intervals (Table - 1).

### Data analysis

Data synthesis was conducted independently for each primary outcome. Publication bias was checked using the Funnel plot and Egger’s test [33] and Duval and Tweedie’s Trim and Fill analysis was conducted to correct any identified publication bias [34]. The percentage of Heterogeneity among studies was checked by *I*^*2*^ statistics, and I^2^ which is greater than 50% was considered as substantial [35]. We used the random effect model to deal with the heterogeneity detected in this review [36]. A meta-analysis of odds ratios and relative risks for adverse birth and infant health outcomes were conducted following a log transformation of the effect sizes. STATA version 14 software was used for data analysis [37]. The protocol was registered in PROSPERO with the registration number CRD42018106714.

## Result

### Study screening process

**Fig. 1:**
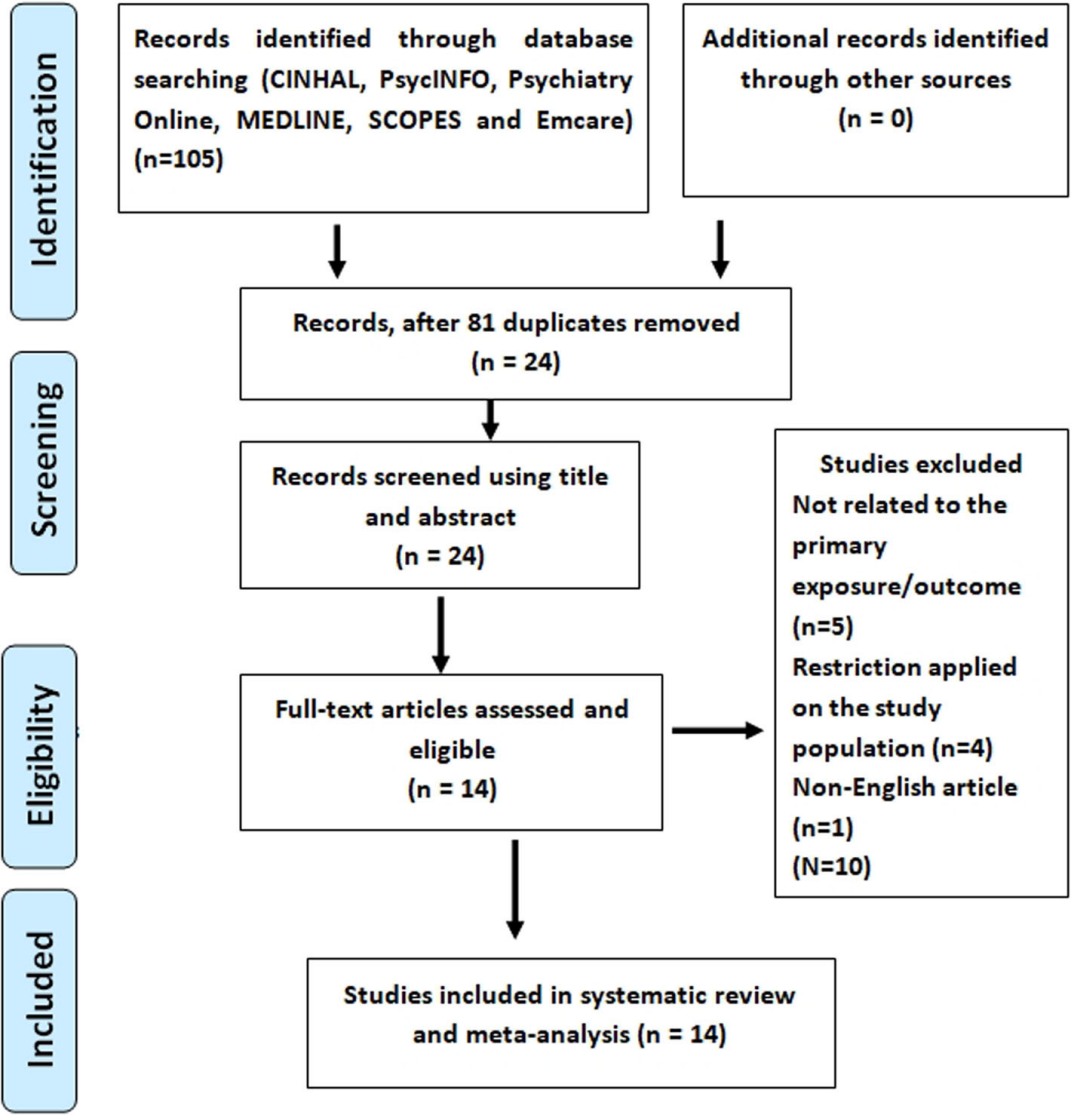
PRISMA flow chart showing study selection for systematic review and meta-analysis of the effect ofperinatal depression on birth and infant health outcomes in Africa, 2007 – 2018

We identified a total of 105 research articles, among which 37 studies were related to the effect of antenatal depression on birth outcomes, and 68 were related to the effect of perinatal depression on infant health outcomes. Following screening 81 duplicates were removed, and from the other 24, ten studies were excluded due to unrelated exposure/outcome (5 studies), restriction on the study population (4 studies), and non-English language use (1 study). Finally, a total of 14 studies: Three for the effect of antenatal depression on birth and 11 studies for perinatal depression effect on infant health were included in the systematic review and meta-analysis (Fig. 1).

### Study characteristics

From the three studies we used to determine the effect of antenatal depression on birth outcomes, we had a total sample size of 1511. These studies were conducted in Ethiopia [38], Kenya, and Ghana [39]. All were longitudinal in study design. The study conducted in Ethiopia was community-based whereas the other two were institution-based studies. The study conducted in Ghana used the Patient Health Questionnaire (PHQ-9) to screen depression whereas the studies from Ethiopia and Kenya used the Edinburgh Postnatal Depression Scale (EPDS).

Among the 11 studies used to determine the effect of perinatal depression on infant health outcomes, we found a total sample size of 22,254. Four studies were from Ghana [40–43] and the other seven studies were from Ethiopia, Kenya [44], Uganda [45], Tanzania [46], Nigeria [47], Zambia [48], and Cote d’Ivoire [40]. Regarding the study setting, six studies were health institution-based and five were community-based. The screening tools used were Diagnostic and Standard Manual for mental Disorder-Revised (DSM-III-R), Self-Reporting Questionnaire (SRQ-20), Patient Health Questionnaire (PHQ), Mini International Neuropsychiatric Interview (M.I.N.I), EPDS, and Center for Epidemiological Studies Depression (CES-D) (Table 1).

**Table 1:** Summary of studies conducted on the effect of postnatal depression on birth and infant health outcome

No	Author, Year	Country, income	Study setting	Study design	Sample size	Time follow up started	Tool used	Type of outcome	RR/OR	LCI	UCI
**Included studies on effect of antenatal depression on adverse birth outcomes**											
[1]	Bindt C et al. 2013	Middle, Ghana	HI	Longitudinal	719	3rd	PHQ-9 > 10	PTB	2.1	0.8	5.6
[2]	Wado WD et al. 2014	Low, Ethiopia	Community	Longitudinal	537	2nd & 3rd	EPDS > 13	LBW	1.77	1.03	3.04
[3]	Mochache K et al. 2018	Kenya, Middle	HI	Longitudinal	255	All	EPDS > 13	PTB	3.8	2.1	4.6
**Included studies on effect of perinatal depression on adverse infant health outcomes**											
[4]	Adewuya AO et al. 2008	Nigeria, Middle	HI	case control	242	6 to 12 weeks	DSM-III-R	Poor weight	3.41	1.3	8.52
								Poor height	3.28	1.03	10.47
[5]	Ndokera R et al. 20,011	Zambia, Middle	Community	Cross sectional	278	2 to 12 months	SRQ-20 > 8	Serious illness	1.64	0.51	5.24
								Diarrhea	1.32	0.71	2.48
								Underweight	1.48	0.35	6.22
[6]	Guo N et al. 2013	Ghana, Middle	HI	Follow up	654	3rd	PHQ > 10	Febrile illnesses	1.32	1.01	1.74
[7]	Guo N et al. 2013	Cote devour, Middle	HI	Follow up	654	3rd	PHQ > =10	Febrile illnesses	1.57	1.2	2.07
[8]	Ashaba Set al 2015	Uganda, Low	HI	case control	166	1 to five yrs	(M.I.N.I.)	Malnutrition	2.4	1.11	5.18
[9]	Weobong B et al. 2015	Ghana, Middle	Community	longitudinal	16,560	4 to 12 weeks	PHQ-9 > 10	Diarrhea	1.8	1.45	2.14
								cough	1.49	1.28	1.7
								Fever	1.8	1.49	2.11
								Vomiting	1.98	1.26	2.71
[10]	Madeghe BA et al. 2016	Kenya, Middle	HI	cross sectional	200	6 to 14 weeks	EPDS > 13	Non-EB	6.14	2.45	13.36
								Under weight	4.4	1.91	11.93
[11]	Wemakor A et al. 2016	Ghana, Middle	HI	cross sectional	384	0–59 months	CED-S > 16	Stunting	2.48	1.29	4.77
[12]	Benett IM et al. 2015	Ethiopia, Low	Community	longitudinal	1885	13 months	CED > 9	Stunting	0.91	0.81	1.02
								underweight	1.01	0.89	1.15
[13]	Neamah H et al. 2018	Tanzania	Community	Follow up	1031	18–36 months	PHQ-9 > 9	Stunting	1.07	0.73	1.56
[14]	Wemakor A et al. 2018	Ghana, Middle	Community	Cross sectional	200	6–23 months	CES-D > 20	Stunting	1.05	0.58	1.91

**Fig. 2:**
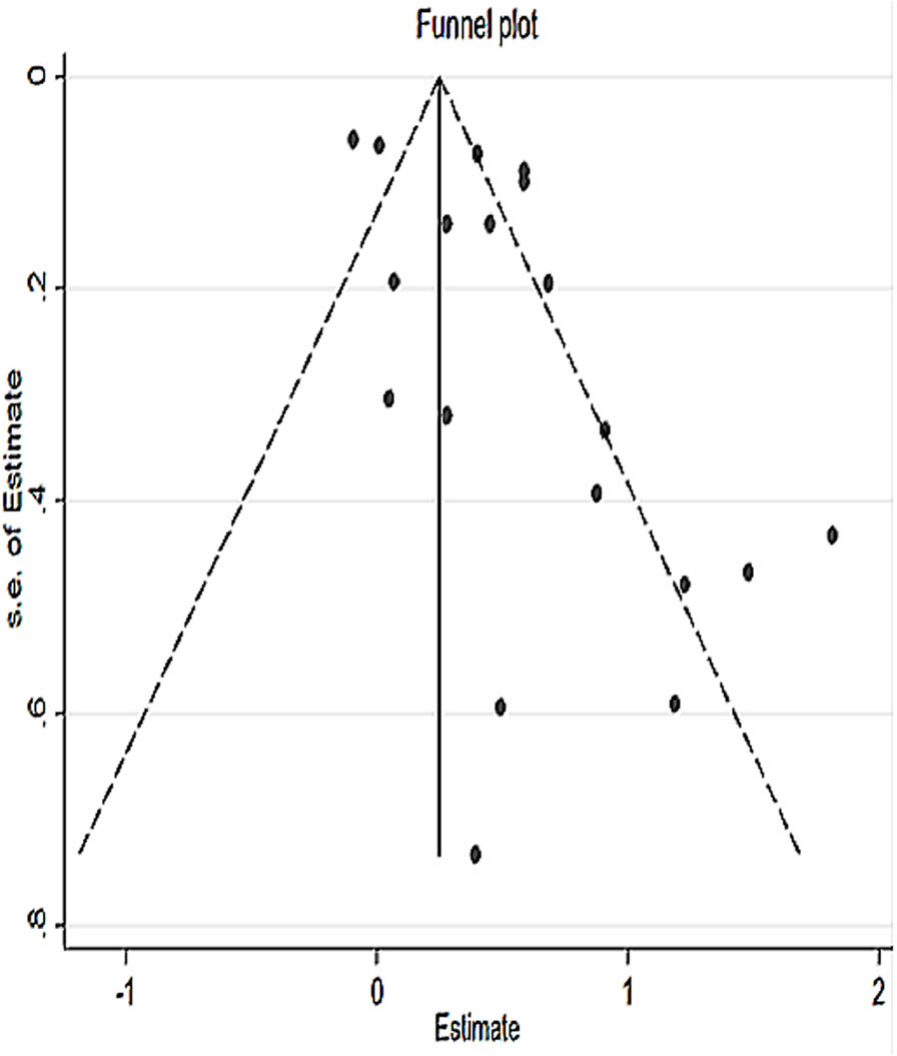
Funnel plot for testing publication bias for systematic review and meta-analysis of the effect of perinataldepression on adverse infant health outcomes in Africa, 2007 – 2018

**Fig. 3:**
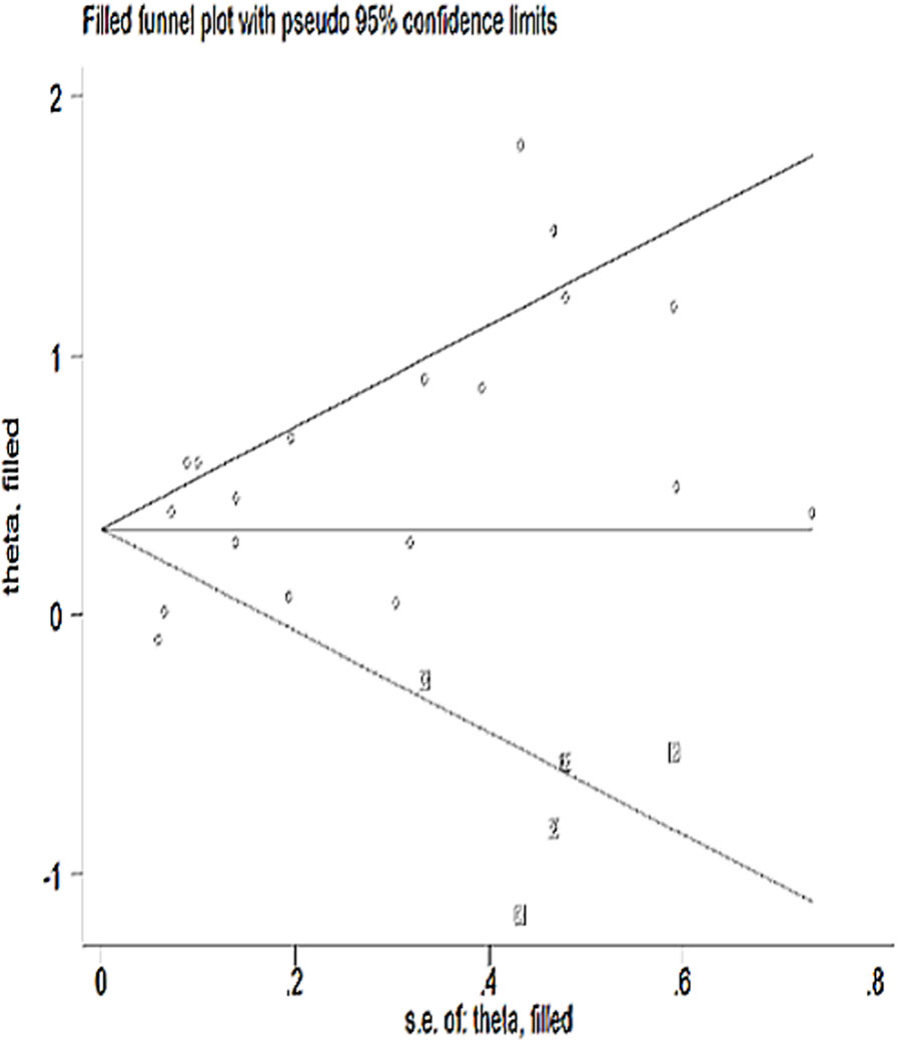
Funnel plot after trim and fill analysis in the systematic review and meta-analysis of the effect of perinataldepression on adverse infant health outcomes in Africa, 2007 – 2018

**Fig. 4:**
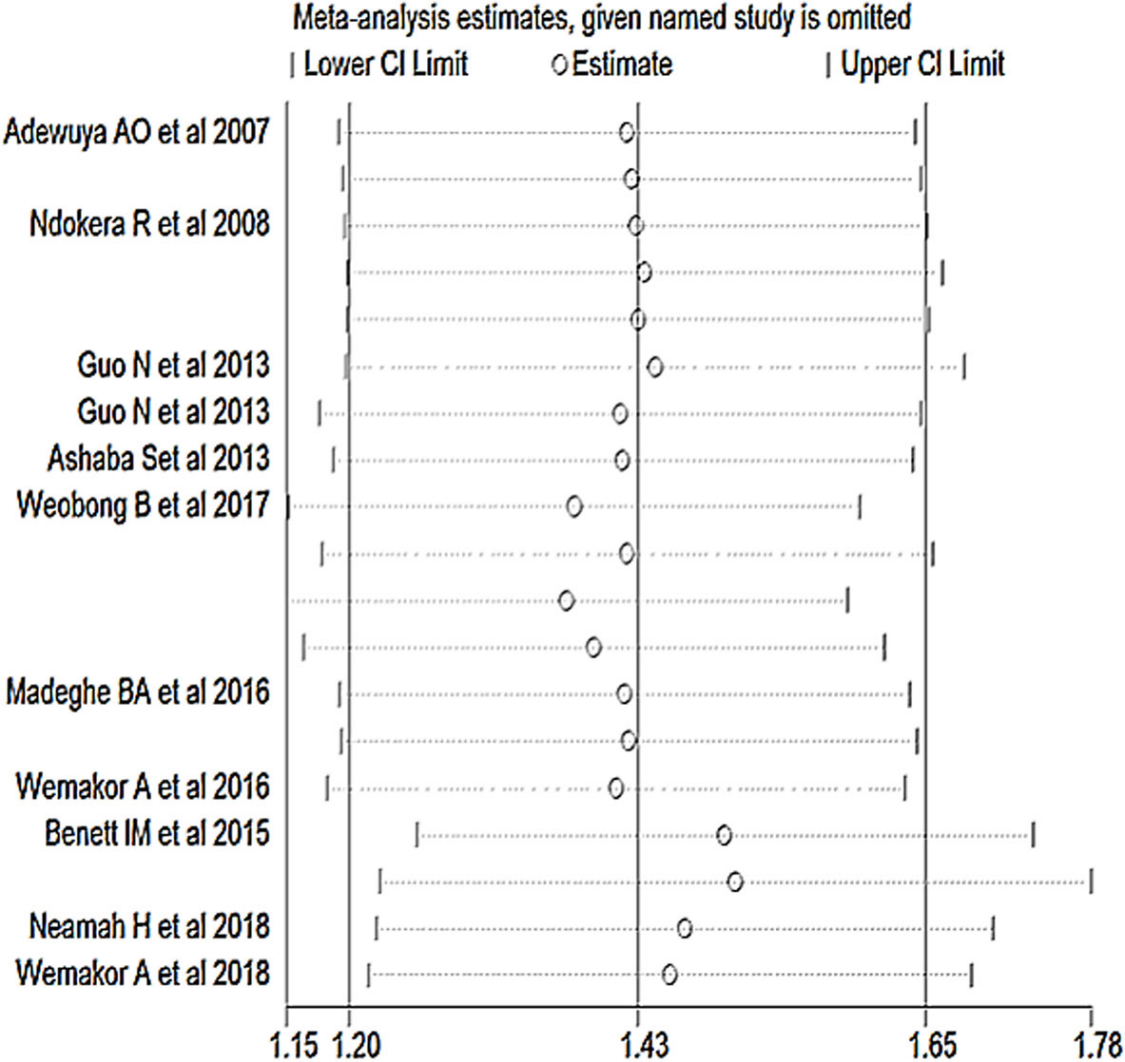
Sensitivity analysis of studies included in the systematic review and meta-analysis of the effect ofperinatal depression on infant health outcomes in Africa, 2007 – 2018

The presence of publication bias was detected by the asymmetrical distribution of studies on the funnel plot (Fig. 2) and a significant egger’s test (*P*-value < 0.05). Therefore, the Duval and Tweedie’s Trim and Fill analysis was used to produce the final pooled estimates (Fig. 3). The presence of any influential study was checked by sensitivity analysis and no study with significant influence on the pooled estimate was observed (Fig. 4).

### Effect of antenatal depression on birth outcomes

**Fig. 5:**
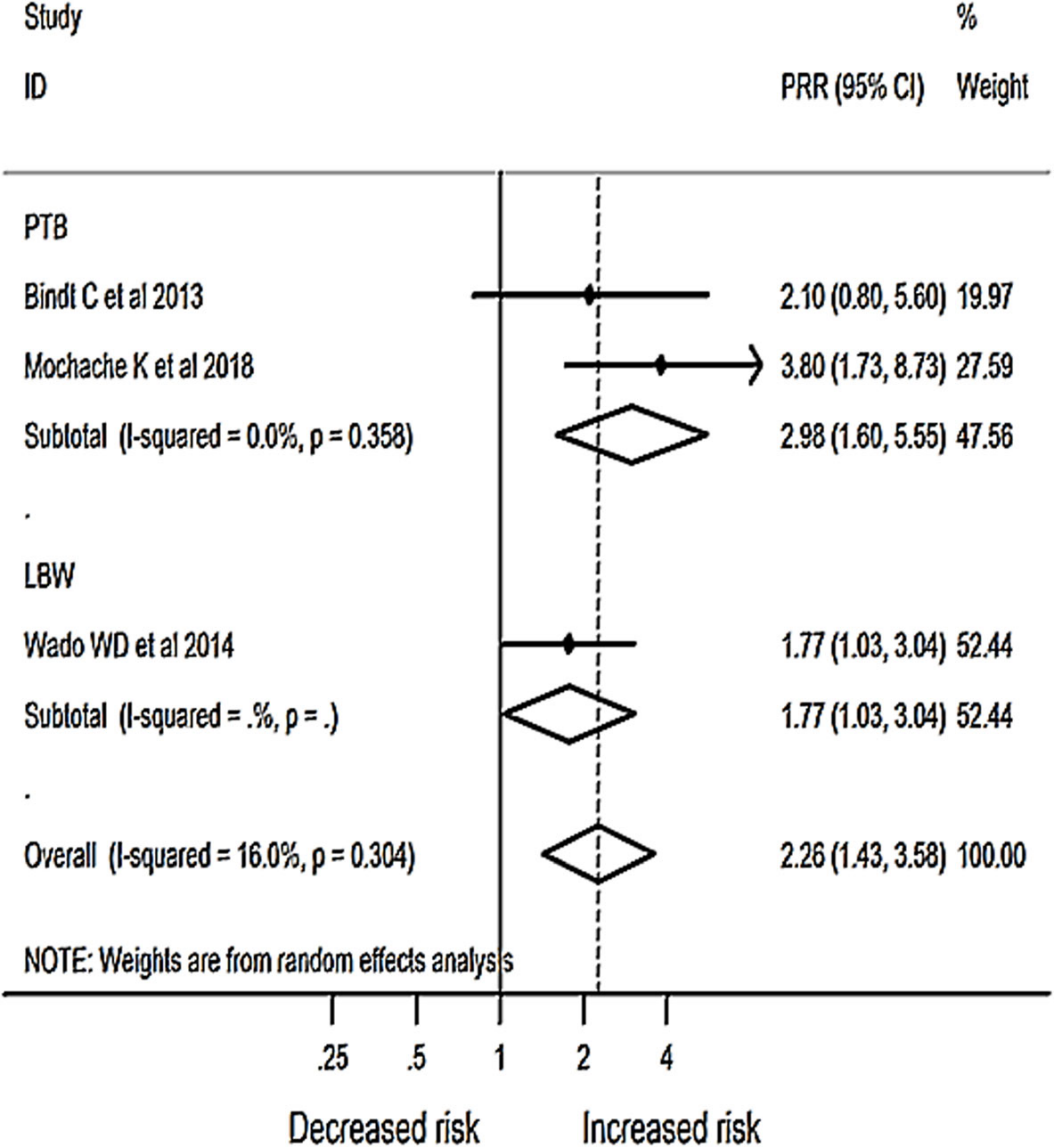
Meta-analysis of the effect of antenatal depression on birth outcome in Africa 2007 – 2018

In this review, we had two studies that showed association between antenatal depression and preterm birth (PTB) with RR of 2.1 and 3.8. The pooled estimate in the random effect model has also shown a similar direction of association between antenatal depression and PTB with a pooled Relative Risk of 2.98 (95% CI: 1.60, 5.55). We had only one eligible study that determined the relationship between antenatal depression and low birth weight that estimated a relative risk of 1.77 (95%CI: 1.03, 3.04). When these three studies are pooled, we found a relative risk of 2.26 (95% CI: 1.43, 3.58), which means a risk of having adverse birth outcomes was 2.26 times higher among mothers who had antenatal depression symptoms than their counterparts (Fig. 5).

### Effect of postnatal depression on infant health outcomes

We conducted a meta-analysis of 11 studies that determined the risk of malnutrition among children born to mothers who had perinatal depression. Among these studies, five found no significant association and six studies showed a positive relationship. The RR in the primary studies ranged from 0.91 to 6.14. Pooling these studies gave a relative risk of 1.65 (95% CI: 1.24, 2.19), which means the risk of being malnourished was 1.65 times higher among mothers with a history of perinatal depression than children born to a mother with no mental depression.

**Fig. 6:**
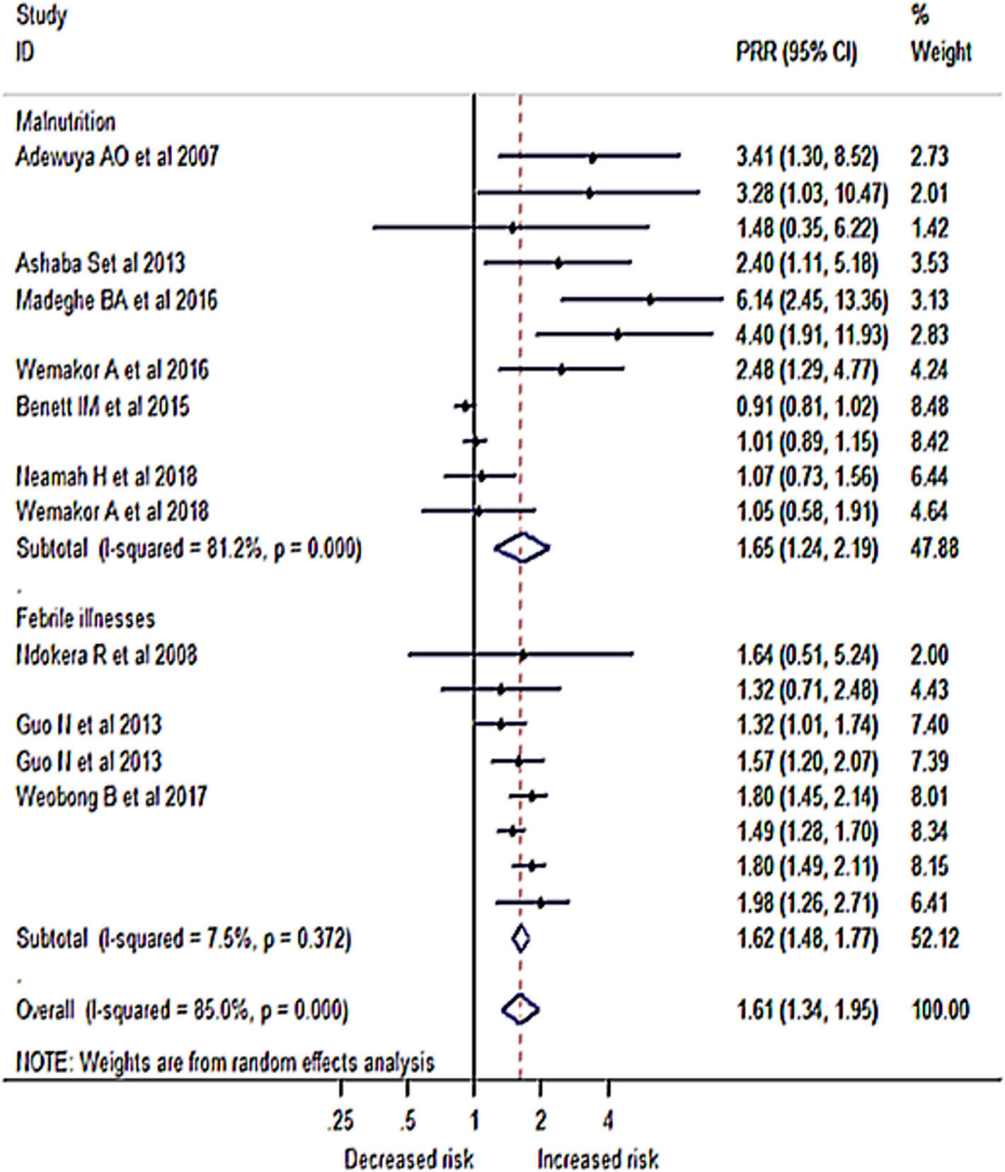
Meta-analysis of the effect of perinatal depression on infant health outcomes sub-grouped by type ofoutcome in Africa 2007 – 2018

A total of eight studies addressed the association between infant illnesses with mother’s perinatal depression exposure; two of them found no significant association but the other studies showed a significant effect of postnatal depression on the occurrence of infant illnesses. The relative risk of the studies ranged from 1.32 to 1.98. The pooled estimate of the association between perinatal depression and infant illnesses was 1.62 (95% CI 1.48, 1.77); meaning the risk of the infant developing illness was 1.62 times higher among mothers who had depression than their counterparts. The pooled estimates from the two adverse infant health outcomes, malnutrition, and infant illness, showed a relative risk of 1.61 (95% CI: 1.34, 1.95) meaning, the overall risk of having adverse infant health outcomes was 1.61 times higher among mothers with perinatal depression symptoms than mothers without perinatal depression (Fig. 6).

Most importantly, the effect of perinatal depression on the risk of adverse infant health outcomes was consistent across studies using screening tools and diagnostic criteria for identifying mothers with depression symptoms. Similarly, the effect of perinatal depression on adverse infant health outcomes was consistent irrespective of study sample size, study design, time of measurement, and estimation techniques used in the primary studies (Table 2).

**Table 2:** Sub-group analysis of the effect of postnatal depression on infant health outcomes in Africa from 2007 to 2018

Variable for sub-analysis	Number of studies	Sample size (N)	PRR, 95%CI, Higgins test
Time of depression measurement			
The first one to six months	5	18,310	1.82 (1.54, 2.15), *I*^*2*^ = 63.2%
The first one to five years	6	3944	1.13 (0.94, 1.35), *I*^*2*^ = 52.5%
Study design			
Longitudinal	5	20,784	1.38 (1.12, 1.69), *I*^*2*^ = 85.2%
Cross sectional	6	1470	2.32 (1.58, 3.40), *I*^*2*^ = 50.5%
Sample size			
< =384	6	1470	2.32 (1.49, 3.60), *I*^*2*^ = 55.7%
> 384	5	20,784	1.42 (1.16, 1.74), *I*^*2*^ = 89.9%
Type of screening tool used			
Diagnostic tool	3	16,968	1.75 (1.54, 2.00), *I*^*2*^ = 31.7%
Screening tool	8	5286	1.40 (1.13, 1.75), *I*^*2*^ = 78.5%
Estimation method			
Odds ratio	5	1192	2.77 (1.72, 4.46), *I*^*2*^ = 58.8%
Relative risk	4	19,754	1.37 (1.09, 1.72), *I*^*2*^ = 89.1%
Hazard ratio	2	1308	1.44 (1.19, 1.74), *I*^*2*^ = 0.0%

*PRR* Pooled Risk Ratio

## Discussion

Depression in pregnancy and the postnatal period in many African countries is not well documented and has not been given attention for intervention due to competing priorities and the belief that it does not immediately cause fatalities. However, its effect is devastating as it affects household income, productivity, child development [49], and quality of life [50]. This review assessed the effect of perinatal depression on birth and infant health outcomes in Africa. The study found that there is a clear relationship between perinatal depression and adverse birth and infant health outcomes that is LBW, PTB, malnutrition, and infant illnesses.

Women with antenatal depression were more likely to have infants with low birth weight. This finding is consistent with previous reviews [13, 17, 51, 52]. The risk of preterm birth was also found to be higher among women with antenatal depression than their counterparts. This association was also supported by other systematic reviews and meta-analyses [53, 54]. The possible reason for these associations could be both biological and/or nutritional. Biologically, the release of stress hormones such as cortisol and catecholamine’s during pregnancy [27] affect fetal growth [19] and gestational age [28]. Nutritionally, depressed pregnant mothers are more likely to have appetite disturbance that could affect their nutritional uptake through which fetal growth can be impaired [29].

Postnatal depression was also found to negatively affect the nutritional status of infants and this finding was in line with previous systematic reviews [55, 56]. This could be due to poor infant feeding practices of mothers who have depression [44], including decreased breastfeeding duration [57], efficacy, and increased breastfeeding difficulty [58]. On the contrary, a review by Surkan and his colleagues reported a lack of association between postnatal depression and infants being underweight [59].

Similar to previous reviews [55, 60], mothers’ exposure to postnatal depression predicted a higher likelihood of infant illnesses. This could be because poorly fed infants tend to be immune-suppressed and are more likely to suffer from infections [53]. Depression has been shown to impact maternal caretaking behavior, which includes bottle feeding and other malpractices that make the infants be exposed to infectious agents [61]. One study reported that a mother who was depressed during mid-pregnancy would have a 30% risk of being unresponsive toward her new baby [62].

The effect of postnatal depression on the adverse infant health outcomes in this study was consistent across studies using screening tools and diagnostic criteria for identifying mothers with depression symptoms. Similarly, the effect of perinatal depression on adverse infant health outcomes was also consistent irrespective of a study sample size, study design, time of measurement, and estimation techniques used in the primary studies.

Since the consequences of perinatal depression are deleterious for both the mother and the baby, prevention of its occurrence should be strengthened. Otherwise, using nonpharmacologic treatments like cognitive-behavioral therapy, supportive psychotherapy, conjoint therapy with the partner should come first to avoid both explicit and implicit side-effects of pharmacologic interventions [63]. However, if the former two approaches are not effective, pharmacologic interventions should be considered.

This review is comprehensive and based on our extensive search it is the first of its kind in Africa. In addition, in our sub-analysis, we have controlled all possible factors that may affect our estimation of the pooled effect sizes. The inclusion of studies published only in the English language and those which measured depression using screening tools with different versions and cut-off values are the limitations of this review. However, we believed that the findings from this review are important and could be used as a highlight to consider perinatal depression as a major public health problem, and thinking the how to address the disorder should be a key message.

## Conclusions

This systematic review and meta-analysis was conducted to determine the effect of perinatal depression on birth outcomes and infant health in Africa. We found a significant association between perinatal depression and adverse birth and infant health outcomes. More importantly, this association was consistent among studies that used a screening tool and diagnostic criteria to identify mothers with depression symptoms. Our findings highlight that it is time to integrate mental health services with other routine maternal health services to improve birth outcomes and reduce infant morbidity.

## Supplementary Information

**Additional file 1.** Example of our search strategy in Pubmed.

## Data Availability

All the data by which the results are based are available in the manuscript.

## Abbreviations

CES-D 20: Center for Epidemiologic Studies depression scale 20
CI: Confidence Interval
DSM-III-R: Diagnostic and Statistical Manual of mental disorders version III
EPDS: Edinburgh Postnatal Depression Scale
HIV: Human immunodeficiency Virus
LBW: Low Birth Weight
MINI: Mini International Neuropsychiatric Interview
NOS: Newcastle-Ottawa Scale
OR: Odds Ratio
PHQ-9: Patient Health Questionnaire-9
PRISMA: Preferred Report Items for Systematic review and meta-analysis
PRR: Pooled Relative Risk
PTB: Preterm Birth
RR: Relative Risk
SRQ-20: Self-Reporting Questionnaire
