# Supplementary material for: Effect of perinatal depression on birth and infant health outcomes: a systematic review and meta-analysis of observational studies from Africa
Source: Arch Public Health. 2022 Jan 20;80:34. doi: 10.1186/s13690-022-00792-8 (PMC8772173; doi:10.1186/s13690-022-00792-8)
Supplement: Supplementary file 1 — Additional file 1. Example of our search strategy in Pubmed. [file 13690_2022_792_MOESM1_ESM.docx]

Example of our search strategy in Pubmed:

|  | *((((((((((((((infant malnutrition) OR poor growth) OR short height) OR non-exclusive breastfeeding) OR exclusive breastfeeding) OR pneumonia) OR fever) OR malaria) OR diarrhea) OR acute respiratory infection) OR low weight) OR short stature) AND Observational Study[ptyp] AND ( "2017/01/01"[PDat] : "2018/08/02"[PDat] ) AND Humans[Mesh] AND English[lang])) AND (((((postnatal depression) OR depression among new mothers) OR depression after birth) OR postpartum depression) AND Observational Study[ptyp] AND ( "2017/01/01"[PDat] : "2018/08/02"[PDat] ) AND Humans[Mesh] AND English[lang]) Filters: Observational Study; Publication date from 2017/01/01 to 2018/08/02; Humans; English; Africa* |
| --- | --- |
